# Fyn–Saracatinib Complex Structure Reveals an Active State-like Conformation

**DOI:** 10.3390/ijms27031143

**Published:** 2026-01-23

**Authors:** Hai Minh Ta, Banumathi Sankaran, Eric D. Roush, Josephine C. Ferreon, Allan Chris M. Ferreon, Choel Kim

**Affiliations:** 1 Verna and Marrs McLean Department of Biochemistry and Molecular Pharmacology, Baylor College of Medicine, Houston, TX 77030, USA; 2 Berkeley Center for Structural Biology, Lawrence Berkeley National Laboratory, Berkeley, CA 94720, USA; 3 Cytiva, Inc., Marlborough, MA 01752, USA

**Keywords:** Fyn, Src-family kinase, saracatinib, AZD0530, dasatinib, X-ray crystallography, surface plasmon resonance, neurodegeneration, kinase inhibitor selectivity, Tau, Tauopathy

## Abstract

Fyn is a Src-family tyrosine kinase implicated in synaptic dysfunction and neuroinflammation across multiple neurodegenerative disorders, including Alzheimer’s disease (AD) and Parkinson’s disease (PD). Saracatinib (AZD0530) is a potent Src-family inhibitor that has been explored as a repurposed therapeutic; however, its clinical utility is limited by poor kinase selectivity caused by high sequence conservation within Src-family ATP-binding sites. Here, we combine surface plasmon resonance (SPR) and X-ray crystallography to define saracatinib recognition by the Fyn kinase domain (KD). SPR single-cycle kinetics shows that saracatinib binds the isolated Fyn KD and full-length Fyn with low-nanomolar affinity, whereas dasatinib binds with subnanomolar affinity and markedly slower dissociation. We determined the crystal structure of the Fyn KD-saracatinib complex at 2.22 Å resolution. The kinase adopts an active-like conformation with the DFG motif and αC-helix in the ‘in’ state and a conserved β3 αC Lys-Glu salt bridge. Saracatinib occupies the adenine and ribose pockets, and engages the hinge through direct and water-mediated hydrogen bonding while complementing a hydrophobic back pocket by van der Waals contacts. Comparison with reported saracatinib-bound structures of other kinases suggests that the active-state geometry observed for Fyn creates a pocket not observed in inactive-like complexes, providing a structural handle for designing Fyn-selective inhibitors. Comparison with all saracatinib-bound kinase co-structures currently available in the PDB (ALK2 and PKMYT1) indicates a conserved monodentate hinge binding mode but kinase-dependent αC-helix conformations, providing a structural rationale for designing Fyn-selective analogues.

## 1. Introduction

Fyn is a brain-enriched Src-family non-receptor tyrosine kinase that regulates synaptic development and plasticity and contributes to neuroinflammatory signaling. Dysregulated Fyn activity has been implicated across multiple neurodegenerative disorders, motivating Fyn as a drug discovery target for CNS indications. A persistent challenge is that the ATP-binding site is highly conserved across Src-family kinases, complicating the design of Fyn selective inhibitors.

Saracatinib (AZD0530) is an ATP-competitive Src-family inhibitor originally developed for oncology and later repurposed to inhibit Fyn in Alzheimer’s disease. Clinical studies demonstrated CNS availability and tolerability, but efficacy has been limited, underscoring the need for next-generation Fyn-directed inhibitors with improved kinome selectivity and binding kinetics [1,2]. As a potency and kinetic benchmark, dasatinib (BMS-354825) is a clinically used Src/ABL inhibitor with high affinity for Src-family kinases and a well-defined binding mode in multiple kinase co-crystal structures [3,4,5].

Fyn plays critical roles in Tau biology. Tauopathies are characterized by abnormal accumulation and redistribution of the microtubule-associated protein Tau (MAPT), and regional Tau burden tracks neurodegeneration and clinical progression [6,7]. Tau can recruit Fyn to dendritic spines via proline-rich SH3-binding motifs, enabling Fyn-dependent phosphorylation of NMDA receptor complexes and downstream excitotoxic signaling; disruption of dendritic Tau-Fyn targeting mitigates amyloid-β toxicity in mouse models [8]. In parallel, Fyn directly phosphorylates Tau at Tyr18, a modification detected in neurofibrillary pathology [9].

High-resolution structural information, together with kinetic measurements, can provide actionable hypotheses for optimizing inhibitor selectivity and residence time. Here, we integrate X-ray crystallography and surface plasmon resonance (SPR) to define saracatinib recognition by the Fyn kinase domain (KD), contextualize the complex through comparison with available saracatinib-bound kinase structures, and benchmark binding kinetics against dasatinib. This work identifies an active-like Fyn conformation and a putative selectivity handle that can be leveraged in structure-guided drug discovery.

## 2. Results

### 2.1. Overall Architecture of the Fyn KD-Saracatinib Complex

**Figure 1:**
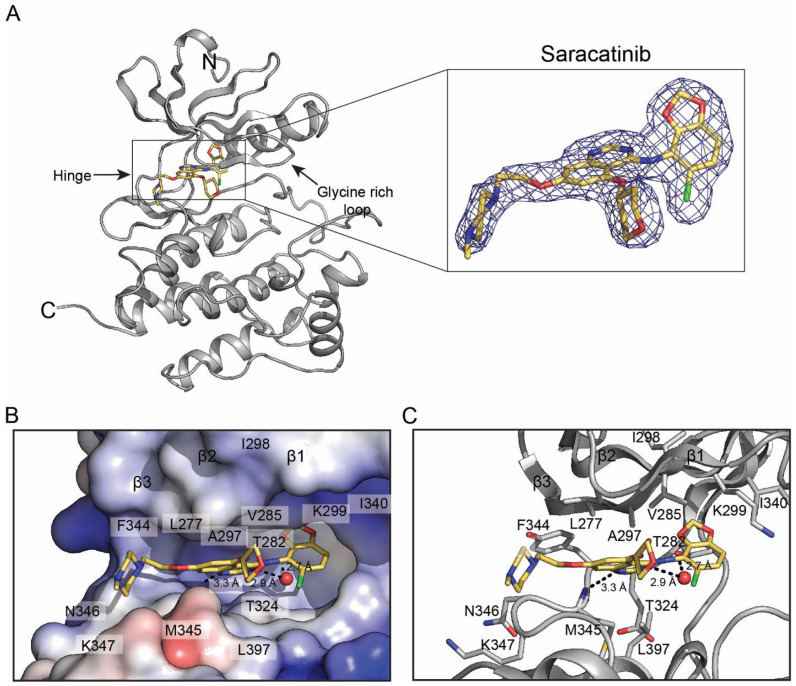
Crystal structure of the Fyn kinase domain bound to saracatinib. (**A**) Overall structure of the Fyn KD-saracatinib complex with 2*Fo*-*Fc* electron density for saracatinib contoured at 1σ (zoom-in-figure). (**B**) Electrostatic surface representation of the active site with bound saracatinib. (**C**) Close-up view of key interactions between Fyn KD and saracatinib. Hydrogen bonds are shown as dashed lines; a water molecule mediating interactions is shown as a red sphere.

To define saracatinib recognition by Fyn at atomic resolution, we co-crystallized the human Fyn KD with saracatinib and determined the structure by X-ray diffraction to 2.22 Å resolution (Table 1). The asymmetric unit contains two Fyn KD molecules with well-resolved electron density for saracatinib in both active sites (Figure 1A). The construct encompasses the catalytic domain (residues 260–537 of human Fyn; UniProt P06241), with only a small number of terminal residues not modeled due to weak density (residues 533–544 including the remaining TEV proteolytic sequence in one molecule are ordered likely due to crystal contacts).

The kinase adopts an active-like conformation with the DFG motif in the ‘DFG-in’ state and the αC-helix in the ‘in’ position, forming the conserved β3-αC Lys-Glu salt bridge (Lys299-Glu314). The glycine-rich loop is in an open conformation, leaving the ribose pocket accessible. Saracatinib occupies the active site cleft between the N- and C-lobes and engages the adenine pocket, ribose pocket, and a hydrophobic back pocket primarily through van der Waals interactions and a small number of hydrogen bonds.

### 2.2. Binding Interactions Between Saracatinib and Fyn KD

The quinazoline core of saracatinib forms a direct hydrogen bond to the backbone amide of Met345 in the hinge, and a water-mediated hydrogen bond to the hinge carbonyl of Thr342 (Figure 1C). The chlorobenzodioxole moiety complements a hydrophobic back pocket formed by Ile298, Lys299, Glu314, and Ile340, whereas the tetrahydropyran group sits in the ribose pocket beneath the glycine-rich loop. The methylpiperazine substituent projects toward the solvent-exposed region near the loop between the hinge and the D-helix and makes contacts with residues in this segment (e.g., Lys347 and Gly348). An electrostatic surface view highlights how saracatinib is accommodated in the active-site cleft (Figure 1B).

### 2.3. SPR Reveals Distinct Binding Kinetics of Dasatinib and Saracatinib to Fyn Constructs

**Figure 2:**
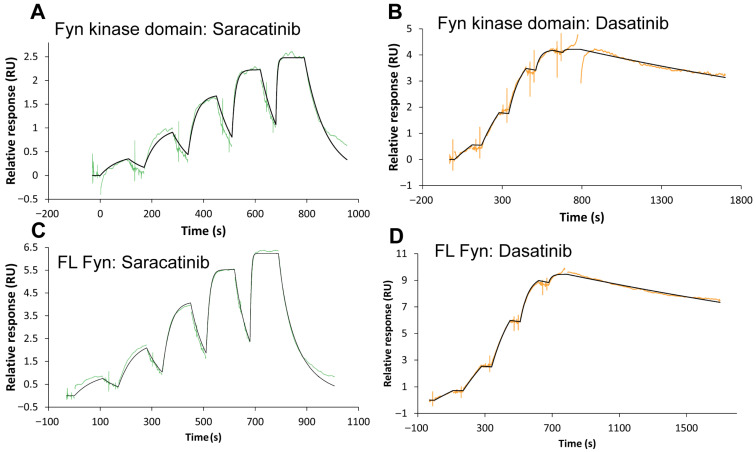
SPR analysis of Fyn kinase interactions with saracatinib and dasatinib. Representative sensorgrams depicting the binding kinetics of saracatinib (**A**,**C**) and dasatinib (**B**,**D**) to the isolated Fyn kinase domain (**A**,**B**) and full-length (FL) Fyn kinase (**C**,**D**). Compounds were injected sequentially at increasing concentrations (five steps), followed by dissociation phases. Experimental data are shown as colored lines, and global fits to a 1:1 Langmuir model are shown in black.

To benchmark saracatinib binding kinetics against a clinically used high-affinity Src-family inhibitor and to inform future structure-kinetics optimization, we quantified binding of dasatinib and saracatinib to recombinant Fyn using single-cycle SPR. Full-length Fyn (Fyn FL) or the isolated kinase domain (KD) was immobilized on an NTA sensor surface using a capture-crosslinking strategy, and five-step concentration series were injected. Representative sensorgrams are shown in Figure 2, and kinetic parameters obtained from global fitting to a 1:1 Langmuir model are summarized in Table 2.

Dasatinib bound both Fyn constructs with sub-nanomolar affinity (K_D_ = 8.93 × 10^−11^ M for KD and 1.40 × 10^−10^ M for full-length), whereas saracatinib bound with low-nanomolar affinity (K_D_ = 1.92 × 10^−9^ M for KD and 6.61 × 10^−9^ M for full-length). The affinity differences were largely driven by dissociation kinetics: dasatinib displayed slow dissociation (k_d_ ≈ 2.8–3.2 × 10^−4^ s^−1^; residence time ~52–60 min), while saracatinib dissociated more rapidly (k_d_ ≈ 2.8 × 10^−3^ to 1.27 × 10^−2^ s^−1^; residence time ~1–6 min) (Table 2).

### 2.4. Active-State Binding Suggests a Fyn-Selectivity Handle

**Figure 3:**
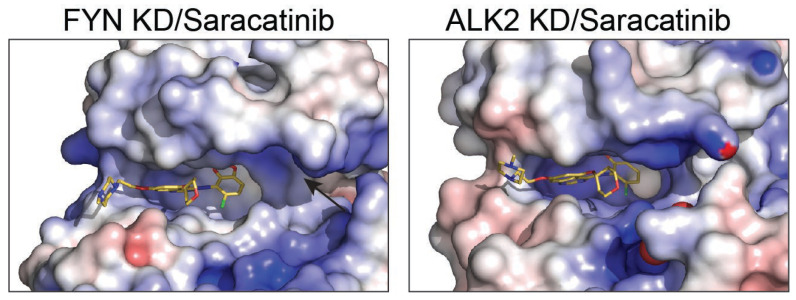
Structural comparison of Saracatinib bound to kinase domains of FYN and ALK2. Surface representation of Saracatinib bound to kinase domains (KD) of FYN (**left panel**) and ALK2 (**right panel**). Electrostatic potential surfaces (negative charge in red, positive charge in blue, neutral in white) illustrate the interaction sites and binding pocket geometries for Saracatinib. The black arrow indicates the αC helix.

Saracatinib has been structurally characterized in complex with other kinases, including Src-family members and the BMP/TGF-β receptor kinase ALK2. In contrast to the active-like Fyn conformation observed here, saracatinib-bound structures of some other kinases have been captured in inactive-like geometries in which the αC-helix is rotated outward and the β3-αC salt bridge is disrupted. We propose that the αC-in arrangement observed for Fyn creates a distinct pocket topology near the αC/β3 region that is less accessible in αC-out complexes (Figure 3). This structural difference provides a hypothesis for improving selectivity: analogs that preserve hinge binding while engaging this additional pocket may bias binding toward Fyn over kinases that preferentially adopt αC-out states in complex with the same scaffold. To systematically contextualize the Fyn KD complex, we compared it to all saracatinib-bound kinase structures currently available in the Protein Data Bank (PDB): ALK2 (6ZGC) and PKMYT1 (5VCX and 5VD3). Across these complexes, saracatinib retains a conserved monodentate hinge interaction to the backbone amide of the hinge residue, whereas water networks and the αC-helix state vary (Table 3 and Table 4). Notably, ALK2 (6ZGC) is captured in αC-out conformations (disrupted β3 Lys-αC Glu salt bridge), whereas Fyn and PKMYT1 adopt αC-in states, suggesting that conformational selection can contribute to kinase-dependent binding kinetics and selectivity. In addition, Fyn and ALK2 exhibit an extra polar contact to a threonine near the hinge/αD region, which may be exploitable for selectivity through appropriately positioned hydrogen-bond acceptors/donors [10,11,12,13]. Polar contacts were identified from deposited coordinates using N/O/S-N/O/S distances ≤3.6 Å. Water-mediated contacts were defined by a water oxygen within 3.6 Å of both ligand and protein heteroatoms. The αC-helix state was approximated by the β3-Lys NZ-αC-Glu OE distance (VAIK/VAVK/YAVK region).

## 3. Discussion

By combining SPR and crystallography, we reveal the molecular details of Fyn’s active-site interaction with saracatinib, which likely underlie its high affinity. Saracatinib and dasatinib exhibit distinct kinetic profiles when comparing the isolated kinase domain to full-length Fyn, suggesting that intramolecular SH3/SH2 domains influence active-site accessibility. Notably, saracatinib dissociates more rapidly from full-length Fyn than from the isolated kinase domain (k_d_ increases from 2.83 × 10^−3^ to 1.27 × 10^−2^ s^−1^; Table 2), consistent with SH3/SH2-mediated regulation reducing stability of the bound state.

The Fyn KD-saracatinib structure reveals a canonical ATP-competitive binding mode: saracatinib anchors to the hinge region, occupies the hydrophobic back pocket, and stabilizes an active-like αC-in/DFG-in conformation. This geometry contrasts with the inactive conformations commonly observed for Src-family kinases in complex with type I½/II inhibitors and provides a structural snapshot of how saracatinib can engage the catalytically competent state (Figure 2 and Figure 3).

Selectivity within Src-family kinases remains challenging because the ATP pocket is highly conserved. Nevertheless, the active-state complex highlights a region proximal to the αC helix and activation loop where modest sequence/shape differences among Src-family members could be exploited for higher selectivity. We propose that functionalization of the solvent-exposed heteroaryl/amide region to interrogate this pocket could provide a “selectivity handle” for Fyn over closely related kinases (Figure 3).

Table 3 provides a cross-kinase interaction map for saracatinib and indicates that, despite diverse kinase folds, the dominant conserved anchor is a single polar contact between the quinazoline core and the backbone amide of the hinge residue. In contrast, the presence/absence of ordered waters and the αC-helix state differ substantially across kinases, with αC-in complexes (Fyn, MST3, PKMYT1) maintaining the β3 Lys-αC Glu salt bridge and αC-out complexes (c-Src, ALK2) exhibiting a disrupted salt bridge. These observations suggest that adding functionality that either (i) stabilizes αC-in binding or (ii) sterically/electrostatically penalizes αC-out geometries could be an effective route to improve selectivity.

For Fyn specifically, the solvent-front region and the loop connecting the hinge to the αD helix (including Lys347 and Gly348) are positioned to be accessed by substituents projecting from the solvent-exposed methylpiperazine exit vector. Introducing an additional ‘secondary anchor’ (e.g., a hydrogen-bond acceptor/donor or an ion-pairing element) to engage this region could simultaneously increase Fyn selectivity within the Src-family and reduce the dissociation rate (k_d_), thereby improving residence time. Such modifications are expected to be better tolerated for saracatinib than for more tightly packed scaffolds, because the piperazine is largely solvent-exposed in the Fyn KD complex (Figure 1B).

The SPR kinetics are also consistent with differences in polar anchoring between dasatinib and saracatinib. In representative dasatinib-kinase co-structures (e.g., c-Src 3G5D, ABL1 2GQG, and Lyn 2ZVA) [3,4,5], dasatinib forms a bidentate hinge interaction (contacts to both the hinge backbone amide and carbonyl) and an additional direct polar contact to a gatekeeper threonine. By contrast, saracatinib typically forms a monodentate hinge contact and relies more heavily on water-mediated interactions (Table 3). A stronger multi-point polar network is expected to raise the energetic barrier to dissociation and primarily decrease k_d_ rather than k_a_, which may explain the markedly slower off-rates observed for dasatinib relative to saracatinib (Table 2) [14,15].

Fyn-selective chemical probes would be particularly valuable for interrogating Tau biology. Tau-driven neurodegeneration is not restricted to AD, and a growing therapeutic focus aims to reduce toxic Tau species and their propagation. Mechanistically, Tau acts upstream and downstream of Fyn: dendritic Tau recruits Fyn to postsynaptic signaling complexes that mediate Aβ-driven synaptotoxicity [8], and Fyn phosphorylates Tau at Tyr18, a modification observed in AD tangles [9]. Our unpublished mass spectrometry data also indicate that Fyn kinase phosphorylates Tau at Tyr18. Thus, Fyn inhibition represents a highly effective strategy to modulate a disease-relevant Tau phosphorylation event while simultaneously disrupting pathogenic synaptic signaling loops that exacerbate Tauopathy.

Clinical repurposing of saracatinib established key translational parameters for Src-family inhibition in the CNS but also revealed the limitations of a broadly acting scaffold. A phase 1b study demonstrated that oral saracatinib achieves measurable CSF exposure at tolerated doses [1], and a phase 2a trial did not significantly slow cerebral metabolic decline over 52 weeks [2]. These data motivate the development of next-generation Fyn-directed inhibitors with improved kinome selectivity, optimized residence time, and brain exposure properties that can be guided by structural insights such as those presented here.

Emerging evidence also implicates Tau phase separation and oligomerization as modulators of Tau toxicity. Our group has contributed to defining how Tau post-translational modifications, electrostatics, and oligomeric states regulate Tau condensation behavior and the emergence of pathological assemblies [16,17,18,19]. Integrating such Tau biophysics with selective Fyn chemical probes may help delineate how Fyn-dependent phosphorylation and synaptic signaling influence Tau condensates and downstream neurotoxicity in cellular models of Tauopathy. Recent work also suggests that Fyn signaling can be modulated by mesoscale organization into nanoclusters/condensates. Tau has been reported to form synaptic nano-biomolecular condensates, and disease-linked Tau condensates can enhance Fyn nanoclustering while favoring an open Fyn conformation, potentially increasing local Src-family signaling. More broadly, biomolecular condensates are increasingly recognized as organizers of cellular biochemistry that can concentrate enzymes and substrates. These observations provide additional biological context for optimizing Fyn-targeted inhibitors: in Tau-rich condensate environments, local concentration effects and rapid rebinding could amplify signaling, placing a premium on both selectivity and favorable binding kinetics for sustained target engagement [20,21,22].

Overall, the kinetic and structural framework reported here establishes saracatinib as a reference ligand for active-state Fyn engagement and provides clear hypotheses for designing improved Src-family selectivity. These insights should accelerate the development of Fyn-selective tools and therapeutic leads for dissecting and potentially modulating Tau-driven neurodegenerative pathways.

## 4. Materials and Methods

### 4.1. Protein Expression and Purification

Baculovirus expression plasmids encoding full-length (FL) human Fyn kinase (residues 1–537) and a Fyn kinase domain (KD, residues 164–493) were generated in pFastBac (Thermo Fisher, Waltham, MA, USA) with an N-terminal 6×His tag followed by a Precision (HRV 3C) protease cleavage site and a C-terminal TEV protease cleavage site followed by GFP and a StrepII tag (His6-3C-Fyn-TEV-GFP-StrepII). Bacmids were prepared using DH10Bac cells, and recombinant baculovirus was produced in Sf9 insect cells. For protein expression, Sf9 cells were infected at a density of 2 × 10^6^ cells/mL and harvested 48 h post-infection by centrifugation, flash-frozen, and stored at −80 °C.

Cell pellets were thawed and resuspended in lysis buffer containing 25 mM Tris-HCl (pH 7.5), 250 mM NaCl, and 1 mM TCEP, and lysed by homogenization. The lysate was clarified by centrifugation and loaded onto Strep-Tactin resin (IBA, Göttingen, Germany). The resin was washed extensively with lysis buffer, and bound proteins were eluted using lysis buffer supplemented with 2.5 mM desthiobiotin.

StrepII eluates were incubated with TEV protease (1:50 *w*/*w*, 4 °C, overnight) to cleave the TEV site between Fyn and GFP-StrepII. The reaction was applied to Ni-NTA resin to capture the His6-tagged Fyn; the resin was washed with lysis buffer and protein was eluted with 250 mM imidazole. To remove the His6 tag, the eluate was incubated overnight at 4 °C with HRV 3C protease (1:50 *w*/*w*) and dialyzed against lysis buffer. After digestion, the sample was again passed over Ni-NTA resin in reverse mode to remove the cleaved His tag, any uncleaved protein, and His-tagged proteases. The flow-through containing tag-free Fyn was concentrated and further purified by size-exclusion chromatography on a Superdex 75 Increase 10/300 GL column equilibrated with same lysis buffer. Peak fractions were pooled, concentrated, flash-frozen, and stored at −80 °C.

### 4.2. Surface Plasmon Resonance (SPR)

SPR experiments were performed on a Biacore 1S+ instrument (Marlborough, MA, USA) using Insight software (v6.0.7). Recombinant full-length Fyn or the isolated kinase domain (both at 30 µg/mL) were immobilized on a Sensor Chip NTA using an NTA-amine capture-crosslinking procedure. Experiments were conducted at 25 °C in PBS-P+ (PBS, with 0.05% (*v*/*v*) Surfactant P20) and 0.2% (*v*/*v*) DMSO). Saracatinib and dasatinib were prepared by serial dilution in running buffer (dasatinib: 0.37–30 nM; saracatinib: 1.2–100 nM) and injected using a single-cycle kinetics protocol at 30 µL/min. Each concentration was injected for 120 s, followed by a 900 s dissociation phase after the final injection. Sensorgrams were reference-subtracted and double-referenced before global fitting to a 1:1 Langmuir binding model to obtain k_a_ and k_d_ values; K_D_ values were calculated as k_d_/k_a_.

### 4.3. Crystallization, Data Collection, and Structure Determination

Purified Fyn KD (1.2 mg/mL) was mixed with a 6-fold molar excess of saracatinib and concentrated to 20 mg/mL. Crystallization trials were set up by hanging-drop vapor diffusion using a Mosquito liquid handler, mixing 250 nL protein-ligand complex with 250 nL reservoir solution and equilibrating against 70 µL reservoir. Crystals appeared after ~3 days in drop using 4.0 M Ammonium acetate, 0.1 M Sodium acetate trihydrate (pH 4.6). The crystals were cryo-cooled using the same solution containing 20% glycerol. Diffraction data were collected at National Synchrotron Light Source II (NSLS-II), beamline 17-ID-2 (Upton, NY, USA). Data were integrated with using XDS (BUILT 20241002) and XSCALE (BUILT 20241002). The structure was solved by molecular replacement in Phaser 2.7.17 [23] using a FYN KD bound with staurosporine (PDB ID: 2DQ7) as a search model, followed by iterative cycles of refinement in PHENIX 1. 21.2 [24] and manual rebuilding in COOT 0.9.8.23 [25]. Model quality was assessed with standard validation metrics, and structural figures were prepared in PyMOL version 2.2.0 [26].

## 5. Conclusions

We report a 2.22 Å crystal structure of the Fyn kinase domain bound to saracatinib and SPR binding kinetics for saracatinib and dasatinib across Fyn constructs. The structure captures an active-like Fyn conformation and reveals specific ligand–protein contacts that explain high-affinity binding. Together, these data provide a structural and kinetic framework for designing next generation inhibitors with improved selectivity for Fyn.

## Figures and Tables

**Table 1:** Data collection and refinement statistics for the Fyn-KD/Saracatnib complex.

Complex	Fyn-KD/Saracatnib
PDB ID code	10DJ
Data collection	
Space group	*P* 2_1_2_1_ 2
Cell Dimensions	
a, b, c (Å)	79.69, 89.60, 92.01
α, β, γ (°)	90, 90, 90
Resolution (Å)	33.85–2.22 (2.28–2.22) ^a^
Rpim	0.1361 (1.148)
CC 1/2	0.991 (0.462)
Completeness (%)	99.69 (99.49)
Multiplicity	8.6 (9.0)
Refinement	
No. of unique reflections	33,168 (2691)
R_work_/R_free_ ^b^ (%)	24.47/26.81 (37.26/40.71)
No. of atoms	
Protein	4332
Ligand	76
R.m.s. ^c^ deviations	
Bond length (Å)	0.003
Bond angles (°)	0.69
Ramachandran plot (%)	
Favored region	97.04
Allowed region	2.41
Outliers	0.56

^a^ Highest resolution shell is shown in parentheses. ^b^ 5% of reflections were excluded for calculation of R_free_. ^c^ R.m.s., root-mean-square.

**Table 2:** SPR-derived kinetic parameters for Fyn interactions with dasatinib and saracatinib.

Immobilized Ligand	Compound	kₐ (M^−1^ s^−1^)	k_d_ (s^−1^)	K_D_ (M)	R_max_ (RU)	Chi^2^ (RU^2^)
Fyn KD	Saracatinib	1.48 × 10^6^ ± 2.16 × 10^4^	2.83 × 10^−3^ ± 3.17 × 10^−5^	1.92 × 10^−9^	2.0	1.39 × 10^−1^
Fyn FL	Saracatinib	1.91 × 10^6^ ± 1.66 × 10^4^	1.27 × 10^−2^ ± 9.83 × 10^−5^	6.61 × 10^−9^	6.6	2.20 × 10^−1^
Fyn KD	Dasatinib	3.56 × 10^6^ ± 4.35 × 10^3^	3.18 × 10^−4^ ± 7.40 × 10^−7^	8.93 × 10^−11^	4.2	2.77 × 10^−2^
Fyn FL	Dasatinib	1.98 × 10^6^ ± 7.01 × 10^2^	2.77 × 10^−4^ ± 2.20 × 10^−7^	1.40 × 10^−10^	9.5	1.16 × 10^−2^

**Table 3:** Structural comparison of saracatinib binding interactions across kinase co-structures in the PDB.

Kinase	PDB ID	Hinge Polar Contact	Additional Direct Polar Contacts	Water-Mediated Polar Contacts	β3 Lys–αC Glu Distance	DFG/DLG Motif Proximity
Fyn	10DJ (this work)	M345(N)–N14 (3.29 Å)	T342(OG1)–O37 (3.54 Å)	N16–H_2_O–T342(OG1)	299–314: 2.88 Å (αC-in)	DFG D408: 4.52 Å
ALK2	6ZGC	H286(N)–N34 (3.15 Å)	T283(OG1)–O26 (3.40 Å); T283(OG1)–O24 (3.44 Å)	—	235–248: 4.53 Å (αC-out)	DLG D354: 5.16 Å
PKMYT1	5VCX	C190(N)–N34 (3.19 Å)	—	N32–H_2_O–T187(OG1)	139–157: 3.06 Å (αC-in)	DFG D251: 3.34 Å

**Table 4:** Sequence comparison of residues in the solvent-front/hinge-αD loop aligned to Fyn Met345-Ser349 (including Asn346, Lys347, and Gly348). Residues are shown for Fyn and other kinases discussed in this study.

Kinase	PDB	KD Sequence Identity vs. Fyn (%)	Met345	Asn346	Lys347	Gly348	Ser349
Fyn	10DJ	100.0	M	N	K	G	S
ACVR1/ALK2 (6ZGC)	6ZGC	26.8	H	E	M	G	S
PKMYT1 (5VCX)	5VCX	22.1	E	L	C	G	P

## Data Availability

Coordinates and structure factors will be deposited in the Protein Data Bank (PDB) under accession code 10DJ. Additional data are available from the corresponding author upon reasonable request.
